# Epicuticular Wax Rice Mutants Show Reduced Resistance to Rice Water Weevil (Coleoptera: Curculionidae) and Fall Armyworm (Lepidoptera: Noctuidae)

**DOI:** 10.1093/ee/nvab038

**Published:** 2021-04-26

**Authors:** Lina Bernaola, Timothy S Butterfield, Thomas H Tai, Michael J Stout

**Affiliations:** 1 Department of Entomology, Louisiana State University Agricultural Center, Baton Rouge, LA 70803, USA; 2 United States Department of Agriculture-Agricultural Research Service, Crops Pathology and Genetics Research Unit, Davis, CA 95616, USA; 3 Department of Plant Sciences, University of California, Davis, CA 95616, USA

**Keywords:** plant–insect interactions, wax mutants, rice, epicuticular wax, host plant resistance

## Abstract

Plant structural traits can act as barriers for herbivore attachment, feeding, and oviposition. In particular, epicuticular waxes (EWs) on the aerial surfaces of many land plants offer protection from biotic and abiotic stresses. In rice (*Oryza sativa* L.), mutations that reduce EWs have been previously reported. However, whether such mutations affect rice water weevil (*Lissorhoptrus oryzophilus* Kuschel) and fall armyworm (*Spodoptera frugiperda* Smith) performance has not been investigated yet. These pests cause significant economic problems in important rice-producing areas of the United States. The aim of our study was to characterize the EWs of EW mutants and wild-type rice plants by gas chromatography–mass spectrometry and compare the resistance of mutant and wild-type plants against rice water weevil and fall armyworm. We hypothesized that mutants with reduced EWs would have weaker resistance to pests than wild-type plants. Three mutant lines (6-1A, 7-17A, and 11-39A) and their wild-type parent (cv. ‘Sabine’) were used to test this hypothesis. Levels of EWs were significantly lower in mutant lines than in the wild-type, and qualitative differences in EW composition were also observed. Reduction in EWs significantly affected performance of insects in experiments conducted under greenhouse conditions. Experiments with rice water weevils were conducted in arenas in which females were given a choice of the mutants and the wild-type for oviposition. Number of first instars emerging from the three EW mutants (an indication of oviposition preference) was higher on the three EW mutants than on wild-type plants with normal wax levels. Similarly, in no-choice experiments using whole plants or detached leaves, weight gains of armyworms on leaves were higher on the mutant lines than on the wild-type. These results indicate that EW traits are involved in rice resistance to weevils and armyworms. Understanding the plant traits that contribute to resistance to rice pests will be helpful for the development of resistant varieties for reducing pest insect damage.

The interactions among plants and their insect herbivores are extremely complex and are important determinants of plant fitness and productivity in natural and managed ecosystems (Stout 2014, Mitchell et al. 2016). On average, herbivores consume approximately 20% of annual net plant productivity (Agrawal 2011), and insect species associated with agricultural crops in particular present an impediment to increasing crop productivity. Currently, management of insect pests relies heavily on the use of insecticides, a strategy that is accompanied by serious challenges, including high costs, nontarget effects on beneficial organisms and humans, and environmental risk (i.e., insecticide residues on surface water; Mitchell et al. 2016, Lowe et al. 2019). In addition, insect pests can overcome, avoid, or develop resistance to insecticides. Therefore, increasing crop productivity by developing more sustainable and cost-effective approaches for preventing crop losses from pests has become an urgent need (Mitchell et al. 2016). Developing sustainable approaches for pest management will require a basic understanding of the mechanisms underlying plant defense and the effects of environmental factors on the expression of those defense-related traits (Mitchell et al. 2016).

The ability of plants to resist or tolerate herbivore attacks contributes to their capability to colonize different environments and maintain productivity in the presence of pest populations (Hanley et al. 2007). Resistance involves the expression of structural or chemical plant traits that deter herbivore feeding, reduce herbivore fitness, or otherwise minimize the amount of herbivore injury to the plant. Plant structural traits such as trichomes, spines, toughness, and waxy cuticles can act as physical barriers to arthropod pest attachment, feeding, and oviposition (Mitchell et al. 2016). The plant cuticle, a hydrophobic layer of fatty acids, alcohols, esters, alkanes, and other chemical compounds deposited on the outer surface of epidermal cells of terrestrial plants, is one structural trait of particular importance in crop protection (Wójcicka 2013, Lewandowska et al. 2020). Waxes are important components of plant cuticles and are found both on the outer surface of, and dispersed within, a lipophilic polymer matrix. The waxes dispersed on the surface of the lipophilic polymer are called epicuticular waxes (EWs; Wójcicka 2015).

Leaf EWs form the outermost protective barrier of the leaf surface of land plants. Although the primary role of EWs appears to be protection from water loss (Schönherr 1976), they are also believed to play important roles in plant resistance against pathogens and insects (Woodhead and Padgham 1988, Eigenbrode 2004, Eigenbrode et al. 2009, Wójcicka 2015, Gorb and Gorb 2017). EWs are among the first resistance-related traits that herbivores encounter upon contact with a plant host and hence serve as an important interface in the interactions of plants with herbivores and the natural enemies of herbivores (van Poecke 2007). Studies with a variety of insect herbivores have shown that EWs often reduce the ability of herbivores to attach to and forage on plant surfaces (Eigenbrode 2004). In addition, chemical compounds found in EWs often exhibit deterrent or toxic effects on herbivores (Hariprasad and van Emden 2010). However, some insects possess adaptations that improve attachment to plants surfaces bearing EWs, and some compounds found dissolved or dispersed in EWs may be phagostimulatory for some insects (Woodhead and Padgham 1988). Thus, the effects of EWs on herbivores are species-specific; in pea, *Pisum sativum* L., for example, reduced waxblooms were associated with lower aphid densities but greater weevil injury (White and Eigenbrode 2000). Similarly, EWs may have both positive and negative effects on predators and parasitoids of herbivores by altering attachment, locomotion, or foraging behavior (Eigenbrode 2004, Eigenbrode et al. 2009). Gorb and Gorb (2017) reported that young waxy culms of several *Bambusa* species, which often show heavy infestations by aphids, hindered, in combination with trichomes, the access of ants to aphid colonies, thereby interfering with the tending of aphids by ants and indirectly protecting the plants from aphids.

Rice, *Oryza sativa* L., one of the main staple crops in the world, is critical to the global supply of energy, protein, fiber, and essential vitamins. In the southern United States, this crop is vulnerable to infestations of several insect pests, which can lead to substantial yield losses. In particular, the most destructive insect pest of rice in the southern United States is the rice water weevil, *Lissorhoptrus oryzophilus* Kuschel (Tindall and Stout 2003, Hamm et al. 2010). The rice water weevil is a leaf- and root-feeding insect that attacks rice fields early in the growing season (Shang et al. 2004). Adults of this species overwinter (diapause) in bunch grasses and leaf litter. Adults emerge from overwintering in spring and fly to rice fields, where they feed on leaf blades of young rice plants, producing long, narrow feeding scars parallel to the venation of leaves. Adult weevils are semi-aquatic and feed on a variety of grassy aquatic weeds in addition to rice. Oviposition is triggered by flooding, and the vast majority of eggs are inserted into leaf sheath tissues at or below the water line (Stout et al. 2002). Larvae that hatch from eggs feed for a short period of time within the aerial portions of the plant before moving down to the roots, where they complete four larval instars on or in the roots. Pupation occurs on roots under flooded conditions; adults that emerge from roots continue feeding on leaves before leaving the field and overwintering in late summer and fall. Adult feeding is not economically important, but larvae have a strong impact on plant yields when they feed on roots of flooded rice (Stout et al. 2002). Another early season pest of rice is the fall armyworm, *Spodoptera frugiperda* Smith. The fall armyworm sporadically infests rice fields in the southern U.S. and causes harm by consuming the aboveground portions of rice plants with its chewing mouthparts. Adult female armyworms oviposit a large number of eggs on leaves, which give rise to larvae that begin to feed on leaves (Stout et al. 2009). Consistently, these two important pests can cause significant economic losses in rice-producing areas when effective control tactics are not deployed.

Levels of rice plant resistance to rice water weevil and fall armyworm have been documented in a wide range of commercial rice varieties under greenhouse or field conditions over the past few decades (Pantoja et al. 1986, Lye and Smith 1988, Stout and Riggio 2002, Vyavhare et al. 2016, Mohamad Saad et al. 2018). These studies have found significant variation in the levels of resistance of rice genotypes to the rice water weevil and/or fall armyworm, but none of these prior studies have investigated associations between resistance and EWs. In rice, mutations that reduce EWs have been known for some time (Yu et al. 2008, Mao et al. 2012, Tai 2015). In a previous study, Tai (2015) reported the identification and preliminary phenotypic characterization of several rice mutant lines exhibiting reduced EWs. However, whether reduced expression of wax in rice affects the performance of rice pests has not yet been investigated. In the present study, we conducted an initial analysis of the mode of inheritance and cuticle wax content and composition of three of these mutant lines. The three mutant lines were chosen for their extreme reductions in EWs as evidenced by scanning electron microscopy of their leaf surfaces and a preliminary characterization of total cuticular wax content (Tai 2015). To determine whether reduced expression of EWs increased susceptibility to insect pests, the resistance of these three EW mutant lines to rice water weevil and fall armyworm was compared with that of the parental wild-type rice variety.

## Methods and Materials

### Characterization of Wax Mutants

Three mutant rice lines, named 6-1A, 7-17A, and 11-39A, with significant reductions in epicuticular waxes were selected for this study. The original mutants were generated by sodium azide seed mutagenesis of the conventional rice cultivar ‘Sabine’, a long-grain tropical japonica, short-season, high-yielding variety developed at the Texas A&M AgriLife Research and Extension Center (Beaumont, TX). The ‘Sabine’ variety is grown in the southern United States and is susceptible to different rice diseases. To confirm the mode of inheritance underlying the three mutants, each mutant (female parent) was crossed with the wild-type progenitor variety ‘Sabine’ (male parent). Resulting F_1_ plants were phenotyped for the wet leaf/glossy (wlg) trait characteristic of the epicuticular wax-deficient mutants as previously described (Tai 2015). F_2_ populations were generated by allowing F_1_ plants to self-pollinate to produce F_2_ seeds that were then planted in a greenhouse.

Cuticular wax composition was characterized by organic solvent extraction followed by gas chromatography–mass spectrometry (GC–MS) analysis. Wax extractions were conducted according to Jung et al. (2006) with modifications as follow. Leaf tissue was sampled from three plants per genotype. Three leaves from each plant were cut 10 cm from the auricle and these leaf blades were then cut into 5 cm lengths and transported from the greenhouse to the lab on ice. Thirty-milliliter HPLC-grade chloroform was placed in 50-ml glass test tubes and heated to 60°C in a water bath. Leaf samples were dipped in chloroform for 30 s to extract the waxes, and leaf blades were temporarily stored postextraction in ultrapure water prior to photographing for determining the leaf surface area. The wax extracts were placed in an N-EVAP nitrogen evaporator (Organomation, Berlin, MA), and N_2_ was applied to remove the chloroform. Wax derivatization was performed by resuspending the dried wax samples in 100-µl chloroform and transferring them to GC vials. Ten-microgram tetracosane (C_24_) from a 1 μg/µl stock was then added to each GC vial as an internal standard. The chloroform was evaporated from samples using a gentle N_2_ stream in the N-EVAP. Thirty-microliter pyridine and 10-µl N, O-bis (trimethylsilyl) trifluoroacetamide (BSTFA) were added to each of the samples which were then incubated at 80°C for 60 min. Pyridine and BSTFA were evaporated using N_2_, and the derivatized wax samples were dissolved in 100-µl chloroform.

Nontargeted profiling of waxes and wax-associated metabolites was performed by GC–MS analysis. Waxes were detected using an Agilent 7890A gas chromatograph equipped with a Gerstel MPS autosampler. Derivatized samples (1 µl) were injected in a 1:10 split ratio. Chromatographic separations were performed on an Agilent DB5-MS column (30 m × 0.25 m × 0.25 μm; Agilent) with helium carrier gas at a flow rate of 1.2 ml/min. The oven program consisted of 80°C held for 30 s, a ramp of 15°C/min to 320°C with a 10-min hold. Inlet temperature was held at 280°C for the duration of the analysis and transfer line was kept at 320°C. Mass spectrometric detection was performed on an Agilent 7000C triple quadrupole mass spectrometer equipped with an Agilent Extractor EI source. The mass spectrometer was operated in scan mode, with data collected between *m/z* 30–650 at a scan time of 250 ms. Control of the GC and MS was performed in Agilent Masshunter v.B05.02, and the MPS was controlled by Maestro v.1.4.12.14.

To identify and quantify metabolites, compounds were deconvoluted in Agilent Masshunter Unknowns v10.0 using a retention index (RI) for an alkane standard (C_7_-C_40_) to convert retention time to mass prior to compound identification using NIST 17. Following compound identification and selection, Agilent MassHunter MS Quantitative Analysis v10.0 was employed to construct an identification method for all samples and for post-evaluation sample processing such as manual integrations. The tetracosane (C_24_) internal standard facilitated comparisons of relative quantities of identified compounds within and between injections.

### Plants and Insects Rearing

For all experiments with insects, four treatments (representing the three *wax* mutants and the wild-type) were employed. Plants of all rice lines were grown individually in 2-liter round (15 cm diameter) plastic pots (Hummert International, Earth City, MO) filled with a sterilized soil mix (2:1:1, soil:peat moss:sand) following a protocol from previous work (Bernaola et al. 2018). Four rice seeds per pot were planted and potted plants were maintained in the greenhouse. Additional fertilizer was unnecessary because these experiments were conducted at early stages of growth.

Adult rice water weevils used to infest plants were gathered from Louisiana State University (LSU) Agricultural Center H. Rouse Caffey Rice Research Station (Crowley, LA, at 30°14′23.4″N latitude and 92°20′46.1″W longitude) rice plots 24 h prior to conducting greenhouse experiments (Bernaola et al. 2018). Collection of rice water weevil occurred in naturally infested areas of this station where insect densities were highest. Weevils were maintained after collection until use in glass jars with freshly cut rice leaves and water. Prior to initiating the experiment, weevils were selected, while they were mating or sexed under a dissecting microscope to guarantee equal numbers of females and males were used in experiments (Bernaola et al. 2018).

Fall armyworm larvae used in greenhouse experiments were sourced from a laboratory-maintained colony. The meridic diet fed to larvae was a commercial formulation for this species in particular (Southland Products Incorporated, Lake Village, AR; Bernaola et al. 2018). Vermiculite-filled buckets were used to maintain pupae. After emerging and mating, females oviposited eggs onto cheesecloth, which was then collected daily and placed in eight-cell trays (Bio-Serv, Frenchtown, NJ). As neonates hatched, they were put into cups containing artificial diet until used in experiments. The colony room was controlled under specific environmental conditions (14:10 [L:D], 28 ± 2°C, 38 ± 2% RH; Bernaola et al. 2018).

### Rice Water Weevil Choice Experiments

To investigate the resistance of *wax* mutant lines to rice water weevil, plants were infested in choice arenas with rice water weevil adults. Two choice experiments were conducted over 2016 (RWW1) and 2017 (RWW2) in a greenhouse on the campus of LSU (Baton Rouge, LA). Large wooden basins lined with heavy black plastic were filled with flood waters to maintain rice under flooded conditions as described in Stout et al. (2002). About 10 d after planting, seedlings were removed to lower the density to two plants per pot. Rice plants were 12–15 d old and had three to four fully developed leaves when experiments were initiated.

To initiate the choice experiments, one pot of each treatment (*t* = 4) was placed into each of seven infestation cages, which served as choice arenas (Supp Fig. 1 [online only]), on the day of infestation. Cages were set in greenhouse basins and basins were flooded to a depth of ~20 cm. Infestation cages consisted of cylindrical wire frames (46 cm diameter × 61 cm tall) covered with a mesh fabric screening (Bernaola et al. 2018). After flooding, adult weevils were released into the flood waters in the centers of the cages at a density of three weevils per plant (24 weevils per cage, equal numbers of males and females) and allowed to randomly feed, mate, and oviposit on the plants in each cage for 5 d. Pots were removed from cages and weevils were discarded (Bernaola et al. 2018). The resistance of *wax* mutants and wild-type plants to rice water weevil was evaluated by recording the numbers of first instars as they hatched and moved to plant roots. Procedures for estimating first-instar densities were adapted from Stout and Riggio (2002). Briefly, after the 5-d adult infestation period, plants from each pot were pulled from soil and washed. Then, each plant was placed in a clean test tube filled with water. Every day, larvae were counted after shaking roots and pouring the water from test tubes into transparent containers for counting (Supp Fig. 1 [online only]). Test tubes were refilled with water before plants were returned back to the tubes. Larvae were counted daily until no additional larvae were found for two consecutive days (Bernaola et al. 2018).

### Fall Armyworm No-Choice Experiments

To investigate whether EWs influence resistance of rice to fall armyworm, weight gains of fall armyworm larvae were monitored while either caged on entire plants or fed excised leaves in petri dishes. A total of three no-choice experiments were conducted in 2016 (FAW1) and 2017 (FAW2 and FAW3). FAW1 and FAW2 were whole-plant experiments conducted in a greenhouse, whereas FAW3 was a laboratory assay conducted using excised leaf material. Plants were grown in the greenhouse as previously described. Approximately 10 d after planting, seedlings in each pot were thinned to three plants. Plants were 3 wk old and possessed three to four leaves when experiments were started.

To initiate the no-choice experiments in the greenhouse (FAW1 and FAW2), infestation cages were placed on 10 plants of each line (Supp Fig. 2 [online only]) on benches. Infestation cages were cylindrical plastic tubes (9 cm diameter × 60 cm tall) with one end inserted into the soil and the top end covered with a mesh-screen lid. Two mesh-covered holes were present on each cage to allow air flow. Second-instar larvae at 4–5 d in age were selected from meridic diet and were stage-synchronized by noting when head capsule began to slip; these larvae were starved for 3 h to void their guts before their initial masses were measured using an analytical balance (model XS105, Mettler-Toledo LLC, Columbus, OH; Bernaola et al. 2018). Larvae with similar initial weight masses (FAW1: 0.018 ± 0.001 g, mean ± SE, *n* = 10 and FAW2: 0.010 ± 0.001 g, mean ± SE, *n* = 10) were used in these experiments. One weighed larvae was released into each cylindrical cage and allowed to feed on leaf material for approximately 8 d. Larvae were observed daily to ensure they were not food limited (Bernaola et al. 2018).

To initiate the no-choice feeding assay in the lab (FAW3), leaf material was cut from greenhouse-grown plants of each line (Supp Fig. 3 [online only]) and placed in 10 petri dishes per line. Larvae were selected and stage-synchronized in the same manner as described above, and larvae with similar weights were chosen before starting the experiment. The feeding assay was conducted in 9-cm-plastic petri dishes lined with moistened cotton batting to maintain turgor in excised tissues following Bernaola and Stout (2019). Larvae were placed with leaf material in petri dishes for 10 d. Leaf material was replaced every 1–2 d to make sure larvae were not food limited.

After ending the feeding experiment in either greenhouse or laboratory, larvae were removed and starved for 3 h to ensure that the larval gut was emptied before final mass was determined and recorded. For each experiment, 10 larvae were used for each treatment for a total of 80 observations for FAW1 and FAW2 and another 40 observations for FAW3.

### Statistical Analyses

Plants in each F_2_ population were classified as mutant or wild-type based on the wlg phenotype, and the segregation ratios were subjected to Pearson’s χ ^2^ test for goodness-of-fit (H_0_: mutant:wild-type = 1:3 for a single-gene recessive mode of inheritance; degrees of freedom = 1; α = 0.01). Statistical analysis of wax composition was performed using the statistical computing R and the R Studio environment. To perform the analyses, the *lm* function was used to fit linear models of a response variable (e.g., alkanes) by genotype; for each model, genotype was defined as the only fixed factor. The *ref.grid* function was used to create a reference grid from each fitted model to enable calculation of least-squares means and inferences using the *lsmeans* function. The *cld* function of the *multcomp* package (*multcomp::cld*) was used to extract information from the linear models required to create a matrix of all pairwise comparisons using the Tukey contrast matrix. From the contrast matrix, statistical groups were surmised using a multiple comparisons test and grouped by compact letter display; an error rate of 0.05 was set as α.

For the insect performance experiments, data were analyzed using SAS 9.4 (SAS Institute Inc 2018). The susceptibilities of rice lines to rice water weevil and fall armyworm were analyzed separately for each experiment by one-way analysis of variance (ANOVA) using a generalized linear mixed model approach (PROC MIXED). For the rice water weevil choice experiments, total number of first instars emerging from a plant was the response variable with treatment as a fixed effect and a set of infestation cages (replication) as a random effect (Bernaola et al. 2018). For the fall armyworm no-choice experiments, weight gain (final weight – initial weight) was the response variable, treatment was the fixed effect, and a set of cages was a random effect. Means were separated using Tukey’s honestly significant difference post hoc test, with α = 0.05.

## Results

### Characterization of Epicuticular Wax Mutants

F_1_ progeny of the crosses of the mutants with the wild-type progenitor ‘Sabine’ were all wild-type (i.e., no water adhesion). The F_2_ segregation ratios for 6-1A/‘Sabine’ (89 wlg:290 wild-type), 7-17A/‘Sabine’ (82 wlg:269 wild-type), and 11-39A/‘Sabine’ (90 wlg:263 wild-type) were consistent with those expected for a single-gene recessive mutation (χ ^2^ = 0.465, df = 1, *P* = 0.4952; χ ^2^ = 0.502, df = 1, *P* = 0.4785; and χ ^2^ = 0.046, df = 1, *P* = 0.8297, respectively, all not significant at α = 0.01).

**Fig. 1.**
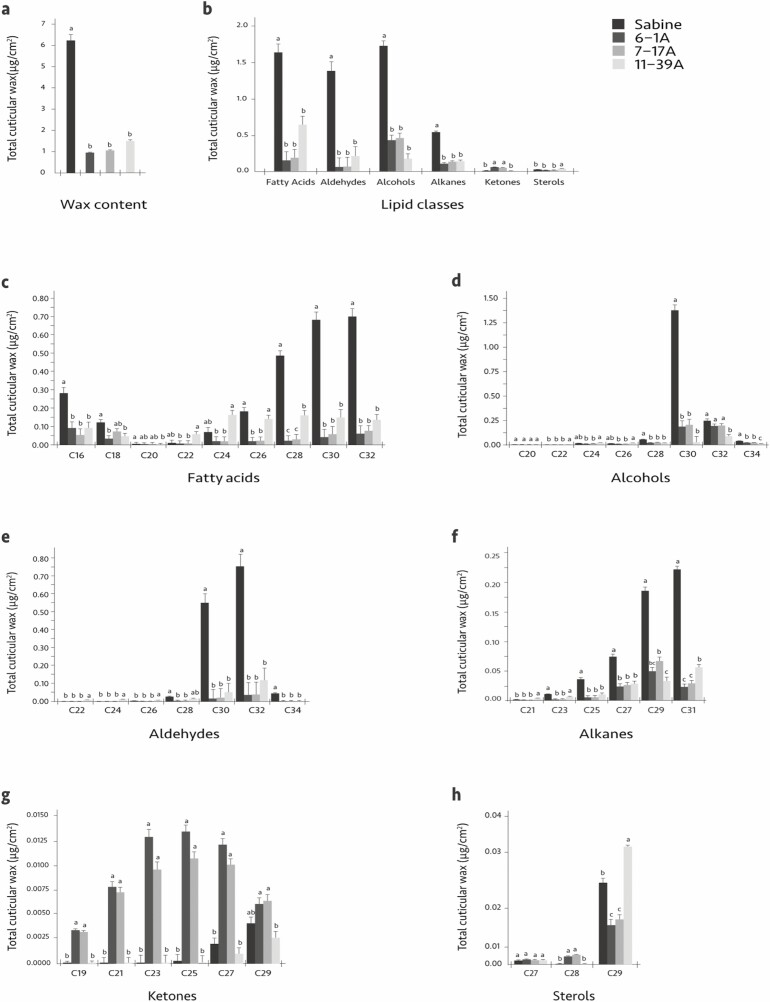
Cuticular wax content and composition in leaves of wild-type and wax mutants (x-axis) analyzed by GC–MS. (a) Total waxes extracted from the surface of rice leaves. (b) Quantification of each lipid class observed from rice leaves. (c–h) Cuticular wax composition and loads of fatty acids, primary alcohols, aldehydes, alkanes, ketones, and sterols from the leaves of the three wax mutants compared with wild-type. Compact letter display indicates significant groups determined by Tukey’s adjustment for each metabolite (α < 0.05).

While fatty acids, alcohols, aldehydes, alkanes, sterols, and ketones were identified, measurable levels of wax esters were not detected. Compared with the wild-type, all three mutants exhibited greatly reduced levels of total waxes, fatty acids, aldehydes, primary alcohols, and alkanes (Fig. 1a–f, Table 1). Fig. 1a shows the reductions in the levels of total wax content of the mutants; these reductions ranged from 76 to 84% (Table 1). Shown in Fig. 1b and Table 1 are the total changes for each lipid class. Shown in Fig. 1c are modifications in the levels of the C_16_-C_20_ long-chain fatty acids (LCFAs) and the C_22_-C_32_ very-long-chain fatty acids (VLCFAs). Total fatty acids were reduced in 6-1A by 89%, 7-17A by 87%, and 11-39A by 64% (Table 1). Changes in primary alcohol levels are shown in Fig. 1d. Total primary alcohols were reduced in the 6-1A mutant by 75%, in 7-17A by 74% and in 11-39A by 90% (Table 1). Reductions in aldehyde levels are shown in Fig. 1e; total aldehydes were reduced in the mutants from 96% in 6-1A, 95% in 7-17A, and 85% in 11-39A (Table 1). Alkane concentrations are shown in Fig. 1f. Alkane concentrations were reduced in the 6-1A mutant by 81%, the 7-17A mutant by 76%, and the 11-39A mutant by 75% (Table 1). Ketone concentrations are shown in Fig. 1g. The levels of total ketones increased in the 6-1A and 7-17A mutants by 627 and 516%, respectively, while the levels of total ketones in 11-39A decreased by 33% (Table 1). Sterol abundance is shown in Fig. 1h. The levels of total sterols in the 6-1A and 7-17A mutants were reduced by 40 and 33%, respectively, while the levels of total Sterols in 11-39A increased by 52% (Table 1).

**Table 1.** Cuticular wax concentration (μg/cm^2^) and percent difference in rice leaf blades

Genotype	Fatty acids	Alcohols	Aldehydes	Alkanes	Ketones	Sterols	Total wax
Sabine	2.64 ± 0.10	1.74 ± 0.11	1.39 ± 0.25	1.62 ± 0.03	0.02 ± 0.00	0.02 ± 0.00	7.44 ± 0.60
6-1A	0.29 ± 0.03 (−88.8%)*	0.43 ± 0.06 (−75.3%)*	0.06 ± 0.01 (−95.9%)*	0.31 ± 0.01 (−80.9%)*	0.17 ± 0.01 (626.6%)*	0.01 ± 0.00 (−39.6%)*	1.28 ± 0.09 (−82.9%)*
7-17A	0.34 ± 0.05 (−87.1%)*	0.46 ± 0.06 (−73.6%)*	0.06 ± 0.01 (−95.4%)*	0.39 ± 0.01 (−75.9%)*	0.14 ± 0.00 (515.8%)*	0.01 ± 0.00 (−32.8%)*	1.44 ± 0.11 (−81.0%)*
11-39A	0.94 ± 0.27 (−64.3%)*	0.17 ± 0.03 (−90.1%)*	0.21 ± 0.09 (−84.7%)*	0.41 ± 0.01 (−74.5%)*	0.02 ± 0.00 (−33.1%)*	0.03 ± 0.00 (51.8%)*	1.79 ± 0.20 (−75.9%)*

*The percent difference (%) was calculated by dividing the difference of ‘Sabine’ and each mutant line by treatment. Wax components were quantified by GC–MS, and the total amount of wax was calculated as the sum of single components. Data represent mean ± SE from three biological replicates.

In the 6-1A and 7-17A mutants, reductions in the fatty acids ranged from 7 to 96%. In the 11-39A mutant, LCFAs were reduced approximately 60%, C_22_-C_24_ VLCFAs were elevated by 54 and 149%, respectively, while C_26_-C_32_ VLCFAs were reduced by 24–81% in comparison to wild-type (Supp Table 1 [online only]). The most abundant primary alcohol in wild-type 'Sabine' is C_30_; decreases of 85–95% were seen in the mutants (Supp Table 2 [online only]). In all three mutants, the most abundant aldehydes, C_30_ and C_32_, were reduced by 91–97% and 84–95%, respectively (Supp Table 3 [online only]). The 6-1A and 7-17A mutants exhibited decreases in alkanes ranging from 54 to 87%. In the 11-39A mutant, the longer chain alkanes were reduced by 42–83% (Supp Table 4 [online only]). The levels of C_19_-C_25_ ketones observed in 6-1A and 7-17A were dramatically elevated (approximately 1,330–3,459% increase). The C_27_ ketone levels of these two mutants were increased by 405–507% relative to wild-type, while the C_29_ ketone levels exhibited more modest increases of around 50%. In contrast, with the exception of C_21_, which was elevated by 32%, the levels of C_19_-C_29_ ketones in the 11-39A mutant were consistently reduced compared to wild-type ‘Sabine’, with reductions ranging from 35 to 50% (Supp Table 5 [online only]). In all three mutants, C_27_ sterols were reduced 7–28%; 6-1A and 7-17A mutants exhibited elevated C_28_ sterols by 21% and 25%, respectively, while C_28_ sterols were unchanged in the 11-39A mutants; C_29_ sterols were decreased in 6-1A and 7-17A by 52 and 46%, respectively, and increased in 11-39A by 54% (Supp Table 6 [online only]).

### Epicuticular Wax Mutants Show Greater Susceptibility to Rice Water Weevil and Fall Armyworm

**Fig. 2.**
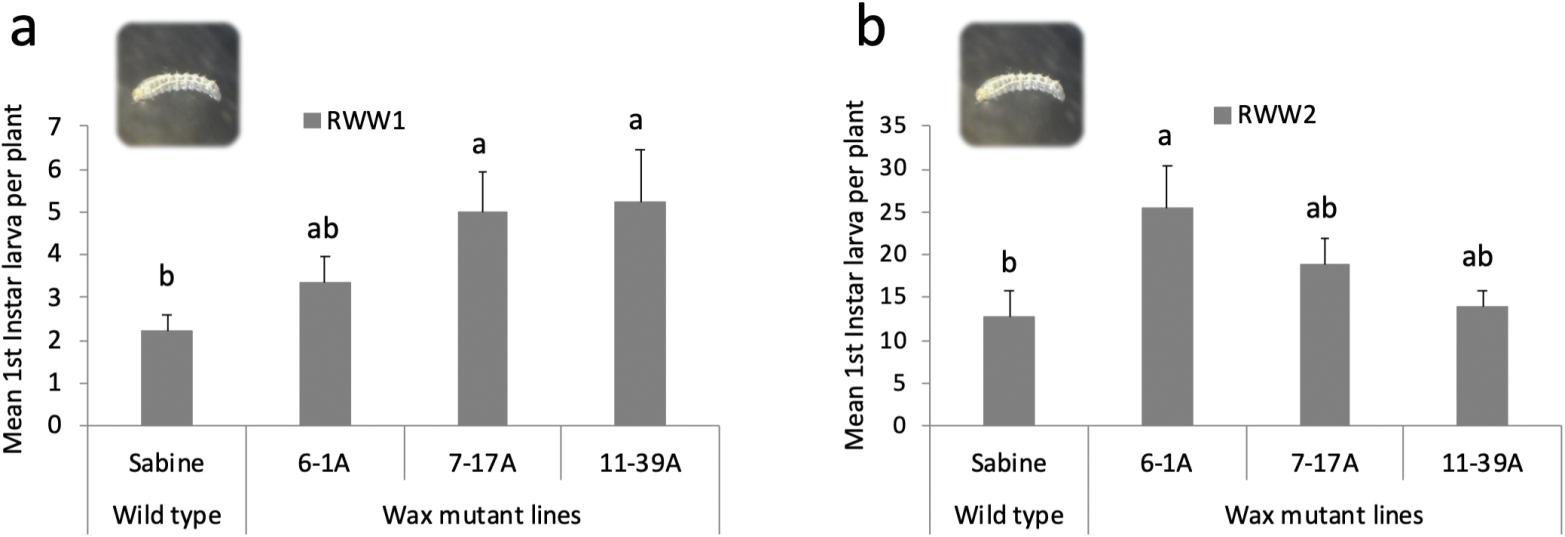
Mean number of rice water weevil larvae emerging per plant (± SE) in two (a and b) greenhouse experiments. Resistance of three epicuticular wax mutant lines (6-1A, 7-17A, and 11-39A) and wild-type ‘Sabine’ rice plants to rice water weevil was investigated by infesting plants with mating couples of weevil adults and allowing them to feed and oviposit on each plant for 5 d, then counting numbers of first instars that emerged from plants. Bars and letters at the column head indicate that means differ significantly (Tukey, *P* < 0.05).

In two independent experiments (RWW1 and RWW2), *wax* mutant plants with reduced EW were significantly more susceptible to rice water weevil (RWW1: *F*_3,28_ = 2.95, *P* = 0.041; RWW2: *F*_3,28_ = 4.60, *P* = 0.010). The numbers of rice water weevil first instars emerging from plants in the two experiments were lower in the wild-type ‘Sabine’ relative to the *wax* mutants (Fig. 2a and b). Increases in emergence of first instars from wax mutants ranged from 51.6 to 135.5% in RWW1 and from 9.6 to 100% in RWW2 (Table 2). Among the mutant lines, 11-39A plants exhibited the highest numbers of first instars in RWW1 (Fig. 2a) and 6-1A exhibited the highest numbers in RWW2 (Fig. 2b).

**Table 2.** Mean number of rice water weevil larvae per plant (± SE) and weight gains (g ± SE) of fall armyworm larvae in two and three experiments, respectively

Genotype	RWW1	RWW2	FAW1	FAW2	FAW3
Sabine	2.214 ± 0.408	12.714 ± 3.160	0.141 ± 0.006	0.109 ± 0.011	0.116 ± 0.011
6-1A	3.357 ± 0.617 (51.6%)*	25.500 ± 4.949 (100.6%)*	0.170 ± 0.006 (20.8%)*	0.137 ± 0.012 (25.2%)*	0.163 ± 0.016 (40.4%)*
7-17A	5.000 ± 0.932 (125.8%)*	18.786 ± 3.187 (47.8%)*	0.175 ± 0.006 (24.1%)*	0.125 ± 0.012 (14.1%)*	0.125 ± 0.007 (7.4%)*
11-39A	5.214 ± 1.223 (135.5%)*	13.929 ± 1.847 (9.60%)*	0.170 ± 0.012 (20.4%)*	0.165 ± 0.013 (51.4%)*	0.157 ± 0.014 (35.0%)*

*The percent difference (%) was calculated by dividing the difference of ‘Sabine’ and each mutant line by treatment.

**Fig. 3.**
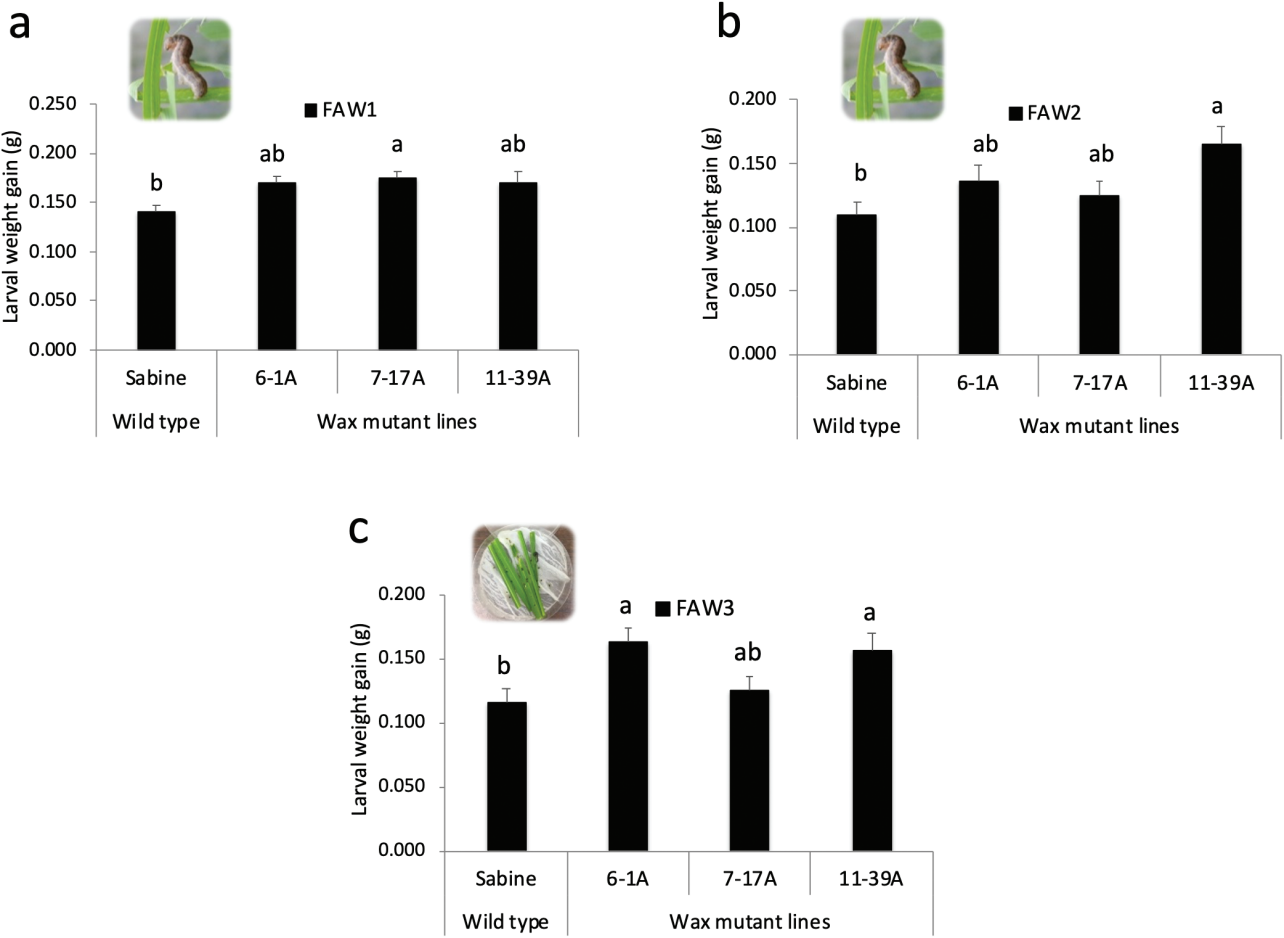
Weight gains (g ± SE) of fall armyworm larvae in two greenhouse experiments (a and b) and one laboratory assay (c). Resistance of three epicuticular wax mutant lines (6-1A, 7-17A, and 11-39A) and wild-type ‘Sabine’ rice plants to fall armyworm was investigated by infesting plants with one armyworm larva on each plant for at least 8 d or by placing armyworms in petri dishes with leaf material for 10 d. Bars and letters at the column head indicate that means differ significantly (Tukey, *P* < 0.05).

Similarly, in the FAW1, FAW2, and FAW3 experiments, the *wax* mutants were more susceptible to fall armyworm (FAW1: *F*_3,36_ = 11.83; *P* < 0.0001; FAW2: *F*_3,36_ = 3.99, *P* = 0.015; FAW3: *F*_3,36_ = 3.23, *P* = 0.024). Weight gains of FAW larvae in the three experiments were lower on the wild-type ‘Sabine’ compared with *wax* mutants. The magnitudes of increases in weight gains on the mutants ranged from 20 to 24% in FAW1, from 14 to 51% in FAW2, and from 7 to 40% in FAW3 (Fig. 3a–c; Table 2). Among the mutant lines, 7-17A plants showed the greatest increases in weight gain in FAW1 (Fig. 3a), 11-39A in FAW2 (Fig. 3b), and 6-1A in FAW3 (Fig. 3c).

## Discussion

In the current study, the genetic basis of the reduced EW phenotypes of the mutants has been confirmed to be the result of single-gene recessive mutations affecting the total cuticular wax composition and content as determined by GC–MS analysis of the cuticle wax components of the leaf. The changes in the wax composition observed in the three mutants are similar to those in other rice cuticle wax biosynthesis mutants (Gan et al. 2016, Wang et al. 2017). Gan et al. (2016) recently reported the cloning and characterization of the cuticle wax-deficient mutant *Oswsl3*, which encodes a β-ketoacyl-CoA reductase. Total waxes were reduced in the *Oswsl3* mutant by 94% in their study. As shown in Fig. 1a, the 6-1A and 7-17A total wax contents were reduced by 83% and 81%, respectively (Supp Table 1 [online only]). Both 6-1A and 7-17A exhibited over 85% overall reductions in fatty acids (Supp Table 2 [online only]), similar to the 95% reduction in fatty acids reported in the *Oswsl3* mutant (Gan et al. 2016). Both the 6-1A and 7-17A mutants exhibited a significant reduction in the C_30_ primary alcohol greater than 85% (Supp Table 3 [online only]), comparable to *Oswsl3* that exhibited a decrease of 99% for this primary alcohol (Gan et al. 2016). The mutant genes in lines 6-1A and 7-17A await identification and characterization.

The wax composition of the 11-39A mutant shows similarity to the wax composition of the *Oswsl4-2* mutant, which harbors a mutation in a β-ketoacyl-CoA synthase (Wang et al. 2017). Both 11-39A and *Oswsl4-2* display a decrease in total waxes, 76% and 83%, respectively (Wang et al. (2017); Supp Table 1 [online only]). Both mutants showed similar reductions in the levels of C_28_-C_34_ VLCFAs, C_28_-C_34_ primary alcohols, and C_27_-C_31_ alkanes (Wang et al. (2017); Supp Table 1 [online only], respectively). Like *Oswl4-2*, 11-39A showed an increase in the levels of C_22_-C_24_ VLCFAs (Wang et al. (2017); Supp Table 2 [online only]). The increases were substantial (541.4 and 149.4% of wild-type, respectively) although not statistically significant. Taken together, the wax composition results for 11-39A suggest that carbon-chain elongation beyond C_26_ is blocked in this mutant. The molecular basis of the altered wax composition and content of these mutants awaits cloning and characterization of the underlying mutations.

The generation and characterization of mutant lines deficient in EWs presented an excellent opportunity to investigate the role of EWs in resistance to insect pests in rice. Prior studies have established that EWs can play important roles in protecting aerial portions of plants from biotic and abiotic stresses (Eigenbrode and Espelie 1995). Because rice water weevil and fall armyworm feed and oviposit on the leaves and leaf sheaths of rice plants and therefore interact with various cues on the surface of plants, such as EWs and lipophilic compounds associated with EWs, we hypothesized that mutants deficient in EWs would exhibit increased susceptibility to insect pests. Our findings support the hypothesis: experiments with three mutant lines, all derived from the cultivar ‘Sabine’, showed that reduced expression of EWs was associated with increased susceptibility to two important insect pests in this crop, the rice water weevil and the fall armyworm. Although there was some variation among experiments in performance of insects on the wild-type and three mutant lines, the overall increases in insect performance (as measured by numbers of first instars or weight gains) were significant in all five experiments.

A positive association of EWs with rice resistance to insect pests is consistent with a large body of literature showing that EWs contribute to plant resistance by influencing insect attachment and mobility, feeding, and oviposition (Eigenbrode and Espelie 1995, Lewandowska et al. 2020). In rice, Woodhead and Padgham (1988) presented evidence that increased activity (reduced settling and probing after exploration) of the brown planthopper, *Nilaparvata lugens*, on a resistant rice variety was attributable to carbonyls and hydrocarbons in the EWs of the resistant variety. Uematsu and Sakanoshita (1989) and Hariprasad and van Emden (2010) demonstrated that the presence of a wax bloom on leaves of cabbage, *Brassica oleracea*, discouraged oviposition and feeding by diamondback moth, *Plutella xylostella*. The ability of the mustard beetle, *Phaedon cochleariae*, to adhere to leaves of *B. oleracea* was reduced on glaucous relative to glossy lines. However, EWs are not always positively associated with plant resistance, and in fact the role of EWs in resistance can differ for different herbivore species on the same plant (Canassa et al. 2021). In experiments involving seven genotypes of pea, *P. sativum* L., White and Eigenbrode (2000) showed that damage to leaves and stipules by leaf weevils (*Sitona lineatus*) was greater on peas with reduced waxblooms, but complicating this relationship is that densities of aphids (*Acyrthosiphon pisum*) were lower on the reduced waxbloom genotypes.

Plant surfaces with wax coverage may also alter attachment, locomotion, and foraging behavior of insect predators and parasitoids (Eigenbrode 2004). For example, EWs are a primary factor determining attachment by the predator *Hippodamia convergens* to *Brassica* leaves (Eigenbrode and Jetter 2002). Eigenbrode et al. (2009) reported that larval and adult coccinellids showed significantly better attachment and higher consumption rates of aphids on leaf surfaces of pea mutants with strong wax coverage compared with those with reduced-wax coverage.

The assay employed in this experiment to evaluate susceptibility of rice to the rice water weevil involved presenting mated female weevils with reduced EW mutants and wild-type ‘Sabine’ in a choice arena and counting the number of first instars emerging from plants subsequent to exposure and oviposition. Although potential effects of EW on the survival of rice water weevil eggs or early instars cannot be completely ruled out by these experiments, the most likely explanation for the increased numbers of first instars on reduced-wax mutants is that chemical cues associated with EWs interfered with or influenced some aspect of weevil oviposition. It may be that EW chemical composition influenced weevil behavior during the process of oviposition. As already noted, chemical cues associated with rice surface waxes have been shown to influence feeding behavior of *N. lugens* (Woodhead and Padgham 1988), and resistance in other rice varieties and lines to the rice water weevil appears to result at least partly from interference with oviposition (Mohamad Saad et al. 2018). Potential effects of EWs on rice water weevil attachment and movement on plants were probably less important in these experiments, because the vast majority of rice water weevil oviposition takes place below the water line (Stout et al. 2002), where the insect is supported by the buoyancy of water. However, more detailed work will be needed to determine what components of rice EWs may be affecting oviposition behavior in the rice water weevil, because the EWs of the reduced-wax mutants differed both quantitatively and qualitatively from the EWs of the wild-type. Additional experiments will also be needed to definitively exclude the possibility that survival of first instars was affected by differences in EWs.

The assay employed to evaluate resistance of rice lines to the fall armyworm showed that growth of larvae was higher on rice lines with reduced expression of EW than on the wild-type ‘Sabine’, but again provided little information on potential mechanisms responsible for greater growth on reduced-wax plants. The reduced-wax mutants used in these experiments differed from the wild-type not only in the total amounts of EWs expressed, but also in the composition of the EWs (e.g., the mutants expressed a relatively higher proportion of ketones in their EWs). Thus, greater weight gains of fall armyworm larvae on mutant lines may have resulted from reduced expression of deterrent or growth-reducing compounds on mutant lines, leading to higher consumption and growth rates, respectively. Alternatively, EWs in the mutant lines may have contained a relatively higher proportion of phagostimulatory compounds. Differences in the chemical composition of EWs on a resistant glossy cabbage variety were implicated in resistance-related changes in the feeding behavior of larvae of the diamondback moth, *P. xylostella*, by Eigenbrode et al. (1991). In addition to alterations in feeding behavior on reduced-wax relative to wild-type rice, increased ability of fall armyworm larvae to attach to, and move on, leaves of reduced-wax mutants may have also contributed to increased growth on these mutant lines, at least in the whole-plant experiments (FAW1 and FAW2). Further experiments will be needed to elucidate the mechanisms responsible for increased fall armyworm growth on reduced-wax lines.

In summary, we demonstrate, for first time, that EWs likely contribute to rice resistance to two major insect pests, although the mechanisms by which EWs contribute to resistance remain to be explored. In addition, EWs are known to have diverse effects on plant pathogens (Lewandowska et al. 2020), and the effects of these wax mutants of wild-type ‘Sabine’ on important pathogens of rice should be investigated. Insect herbivores and pathogenic microorganisms severely affect the growth and yields of rice as well as other crops, and the breeding of crops with resistance to herbivores and pathogens has become an urgent need. Understanding the role of EWs in the resistance of rice to herbivore and pathogens may facilitate efforts to breed lines more resistant or tolerant to insect and pathogen pests.

## Supplementary Material

nvab038_suppl_Supplementary_Information
