# Supplementary material for: Epicuticular Wax Rice Mutants Show Reduced Resistance to Rice Water Weevil (Coleoptera: Curculionidae) and Fall Armyworm (Lepidoptera: Noctuidae)
Source: Environ Entomol. 2021 Apr 26;50(4):948–57. doi: 10.1093/ee/nvab038 (PMC8359818; doi:10.1093/ee/nvab038)
Supplement: nvab038_suppl_Supplementary_Information [file nvab038_suppl_supplementary_information.pdf]

Supplementary Information

Epicuticular Wax Rice Mutants Show Reduced Resistance to Rice Water Weevil (Coleoptera: Curculionidae) and Fall Armyworm (Lepidoptera: Noctuidae)

Environmental Entomology

LINA BERNAOLA[1*], TIMOTHY S. BUTTERFIELD[2], THOMAS H. TAI[2,3], AND MICHAEL J. STOUT[1]

[1] Department of Entomology, Louisiana State University Agricultural Center, Baton Rouge, Louisiana 70803, USA

[2] United States Department of Agriculture-Agricultural Research Service, Crops Pathology and Genetics Research Unit, Davis, California 95616, USA

[3] Department of Plant Sciences, University of California, Davis, California 95616, USA

[*]Corresponding author:

Lina Bernaola, Email: linabernaola@gmail.com

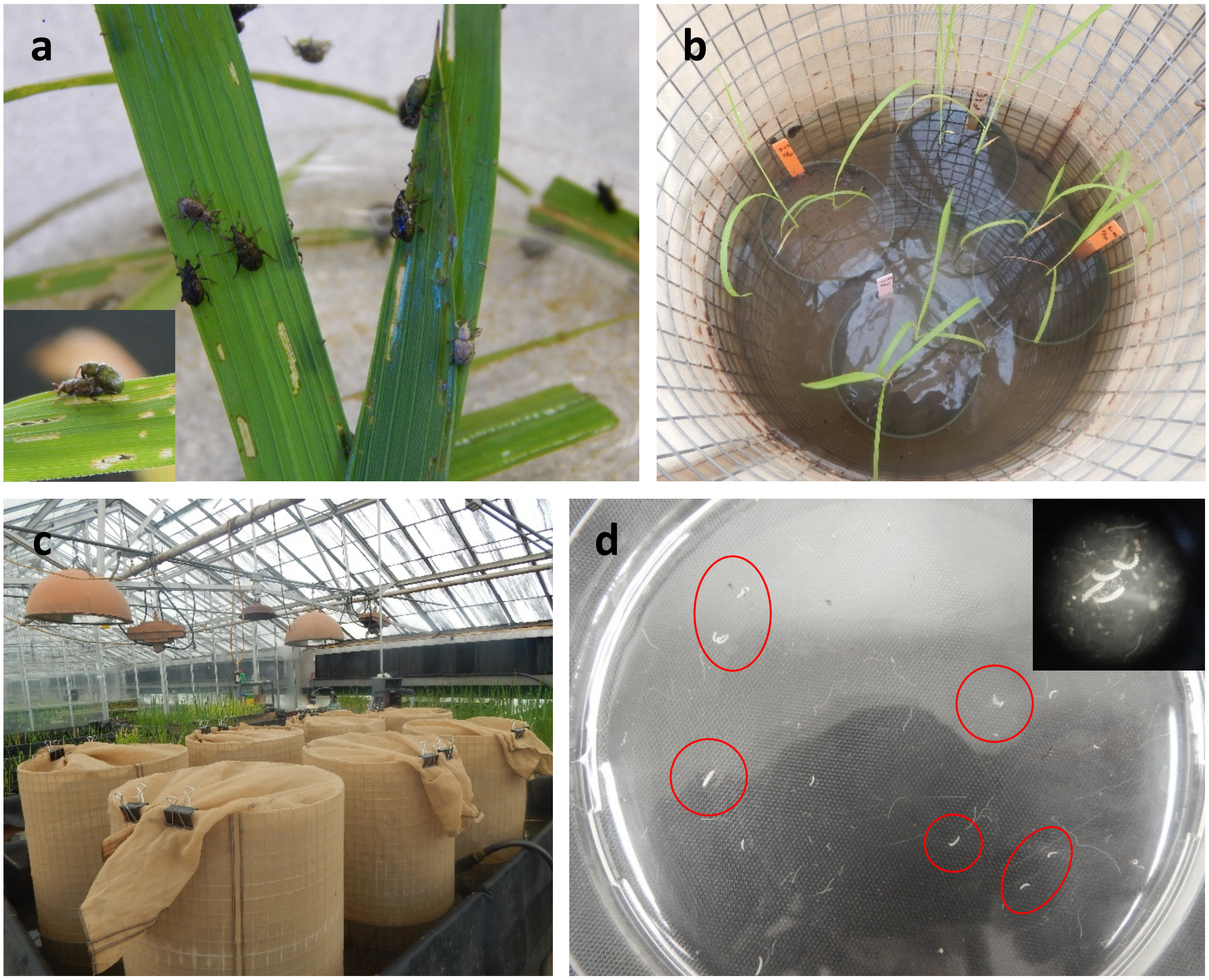

Supplementary Fig. 1 Photographic representation of rice water weevil choice experiment. (A) Adult weevils collected from the field 24 h before the experiment and maintained in jars with rice leaves; (B) Three w*ax* mutants and one wild-type pot were placed in a cage under flooded conditions before weevil infestations; (C) Cages were distributed in the greenhouse basin and basin were flooded to a depth of ~20 cm; (D) Count the number of first instar larvae

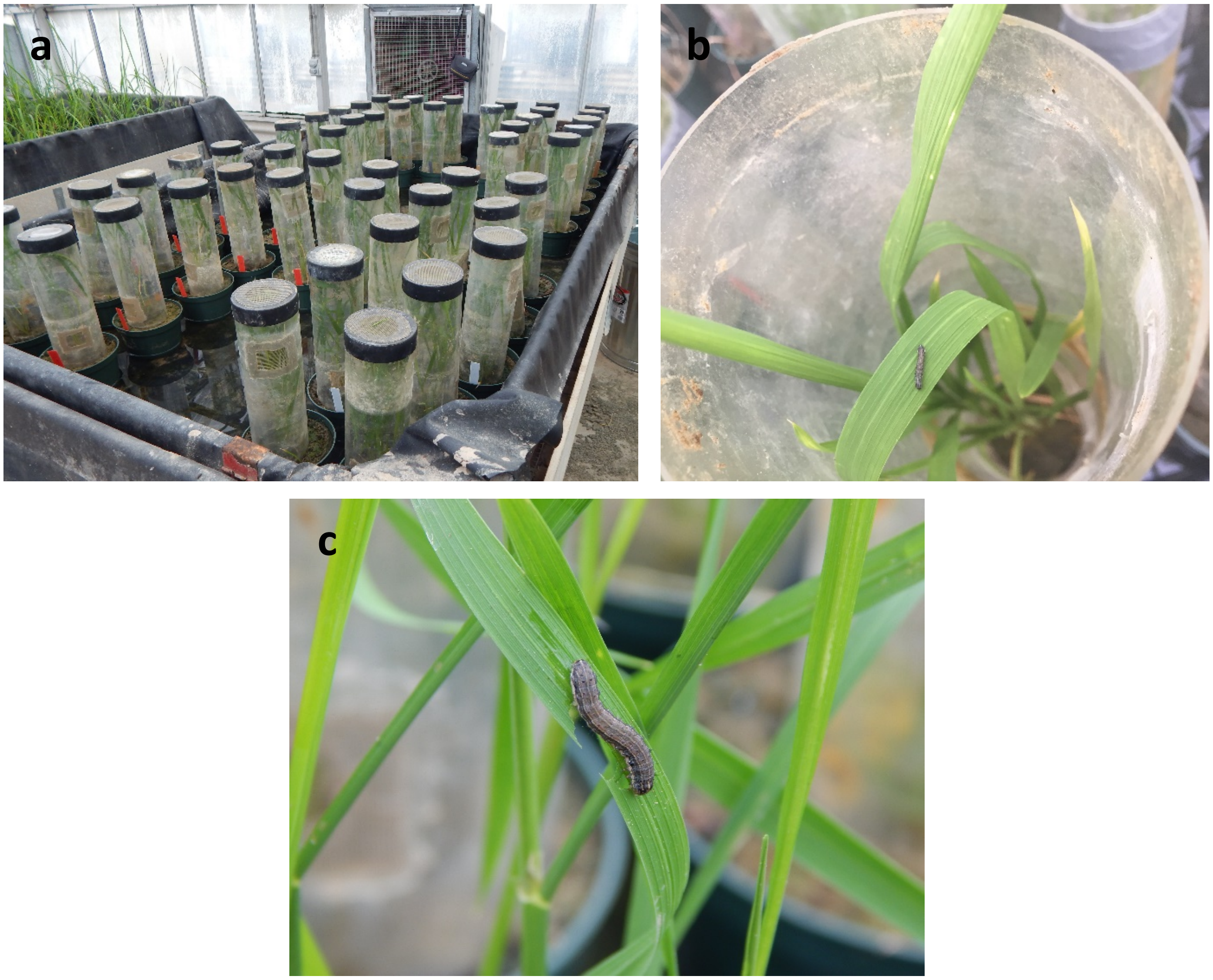

Supplementary Fig. 2 Photographic representation of fall armyworm no-choice experiment. (A) Plants were grown in pots for one month before the experiment and one pot of each line were confined within one plastic cage; (B) One larva of 4-5-d old were selected (from the colony maintained all year around) to initiate the experiment; (C) Larvae were allowed to feed on leaf material for 8 days

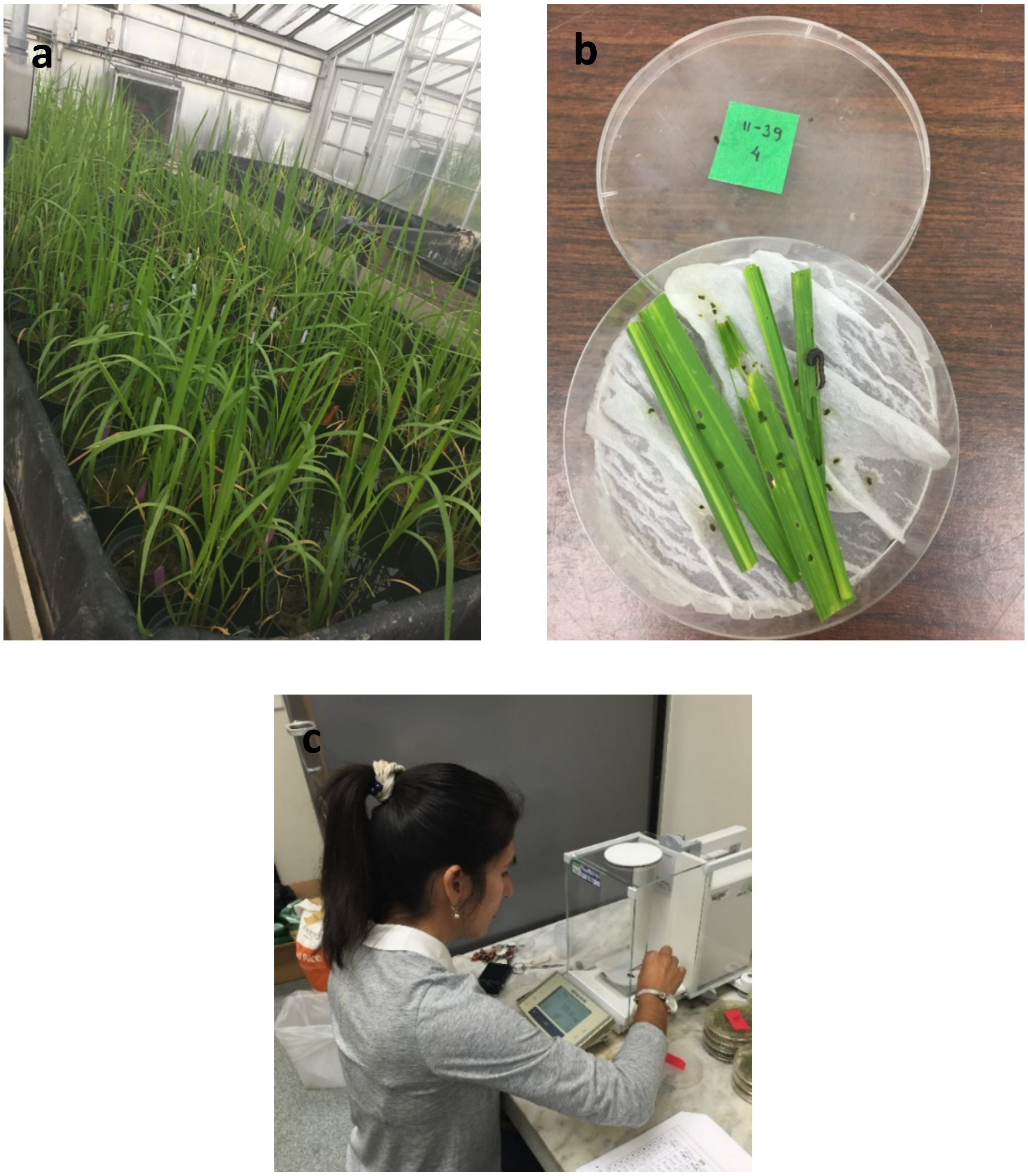

Supplementary Fig. 3 Photographic representation of fall armyworm no-choice experiment for feeding assay. (A) Plants of each line were grown in pots for one month before starting the laboratory assay; (B) One larva of 4-5-d old were selected (from the colony maintained all year around) to initiate the assay; (C) Larvae were allowed to feed on leaf material for 10 days and measured the final weigh

Modified Supplementary Tables, Version 1: Font size 10

Supplementary Table S1 Summary of saturated fatty acids measured in this experiment, three replicates per genotype.

Genotype	C16	SE	Percent Reduction	C18	SE	Percent Reduction	C20	SE	Percent Reduction
Sabine	0.2395	0.0524	0	0.1244	0.009	0	0.0087	0.0010	0
6-1A	0.0904	0.0450	62.26	0.0327	0.002	73.68	0.0057	0.0007	34.28
7-17A	0.0527	0.0105	77.99	0.0717	0.020	42.39	0.0081	0.0013	7.10
11-39A	0.0910	0.0245	61.99	0.0458	0.018	63.20	0.0035	0.0012	60.44

Genotype	C22	SE	Percent Reduction	C24	SE	Percent Reduction	C26	SE	Percent Reduction
Sabine	0.0088	0.0011	0	0.0650	0.0073	0	0.1836	0.0144	0
6-1A	0.0059	0.0008	32.75	0.0186	0.0020	71.34	0.0189	0.0563	89.69
7-17A	0.0068	0.0014	23.36	0.0182	0.0024	72.01	0.0214	0.0028	88.37
11-39A	0.0565	0.0248	-541.4	0.1621	0.0515	-149.4	0.1402	0.0429	23.63

Genotype	C28	SE	Percent Reduction	C30	SE	Percent Reduction	C32	SE	Percent Reduction	Total Fatty Acids	SE	Percent Reduction
Sabine	0.5084	0.0289	0	0.7077	0.0312	0	0.7233	0.0250	0	2.6368	0.0965	0
6-1A	0.0215	0.1773	95.77	0.0410	0.2336	94.20	0.0599	0.2259	91.71	0.2948	0.7631	88.82
7-17A	0.0288	0.0048	94.33	0.0569	0.0095	91.96	0.0752	0.0120	89.60	0.3397	0.0498	87.12
11-39A	0.1598	0.0481	68.56	0.1483	0.0511	79.05	0.1355	0.0542	81.27	0.9426	0.2737	64.25

Supplementary Table S2 Summary of primary alcohols observed; three replicates per genotype.

Genotype	C20	SE	Percent Reduction	C22	SE	Percent Reduction	C24	SE	Percent Reduction	C26	SE	Percent Reduction
Sabine	0.0014	0.0002	0	0.0004	0.0000	0	0.0144	0.0004	0	0.0119	0.0009	0
6-1A	0.0006	0.0000	56.92	0.0002	0.0000	40.66	0.0079	0.0007	45.07	0.0067	0.0007	43.87
7-17A	0.0007	0.0001	49.85	0.0003	0.0000	20.36	0.0102	0.0028	28.87	0.0075	0.0020	36.51
11-39A	0.0014	0.0004	-1.45	0.0019	0.0000	-402.0	0.0193	0.0009	-34.32	0.0157	0.0003	-32.42

Genotype	C28	SE	Percent Reduction	C30	SE	Percent Reduction	C32	SE	Percent Reduction	C34	SE	Percent Reduction
Sabine	0.0528	0.0035	0	1.3781	0.1103	0	0.2452	0.0099	0	0.0373	0.0018	0
6-1A	0.0184	0.0024	65.15	0.1854	0.0248	86.54	0.1914	0.0270	21.95	0.0197	0.0025	47.10
7-17A	0.0219	0.0038	58.50	0.2035	0.0292	85.23	0.1958	0.0218	20.12	0.0196	0.0012	47.55
11-39A	0.0148	0.0002	71.89	0.0250	0.0006	98.19	0.0850	0.0015	65.32	0.0097	0.0000	74.07

Genotype	Total 1° Alcohols	SE	Percent Reduction
Sabine	1.7413	0.1067	0
6-1A	0.4303	0.0579	75.29
7-17A	0.4596	0.0583	73.61
11-39A	0.1728	0.0010	90.08

Supplementary Table S3 Summary of aldehydes observed; three replicates per genotype.

Genotype	C22	SE	Percent Reduction	C24	SE	Percent Reduction	C26	SE	Percent Reduction	C28	SE	Percent Reduction
Sabine	0.0010	0.0003	0	0.0007	0.0001	0	0.0028	0.0006	0	0.0266	0.0063	0
6-1A	0.0005	0.0001	54.78	0.0007	0.0004	2.47	0.0009	0.0002	68.75	0.0028	0.0007	89.38
7-17A	0.0005	0.0002	52.39	0.0002	0.0001	70.57	0.0007	0.0001	75.46	0.0033	0.0003	87.49
11-39A	0.0081	0.0018	-682.1	0.0116	0.0020	-1606.78	0.0056	0.0011	-103.5	0.0140	0.0014	47.27

Genotype	C30	SE	Percent Reduction	C32	SE	Percent Reduction	C34	SE	Percent Reduction	Total Aldehydes	SE	Percent Reduction
Sabine	0.5566	0.1019	0	0.7621	0.1378	0	0.0447	0.0081	0	1.3945	0.2540	0
6-1A	0.0157	0.0034	97.18	0.0359	0.0071	95.30	0.0012	0.0002	97.27	0.0576	0.0116	95.87
7-17A	0.0203	0.0050	96.36	0.0385	0.0066	94.95	0.0011	0.0004	97.50	0.0646	0.0120	95.37
11-39A	0.0509	0.0045	90.85	0.1192	0.0080	84.36	0.0045	0.0006	89.92	0.2139	0.0187	84.66

Supplementary Table S4 Summary of alkanes observed; three replicates per genotype.

Genotype	C21	SE	Percent Reduction	C23	SE	Percent Reduction	C25	SE	Percent Reduction
Sabine	0.0009	0.0002	0	0.0101	0.0018	0	0.0365	0.0055	0
6-1A	0.0004	0.0001	54.45	0.0013	0.0001	87.08	0.0048	0.0005	86.71
7-17A	0.0003	0.0001	62.62	0.0015	0.0003	85.42	0.0050	0.0013	86.26
11-39A	0.0033	0.0005	-263.9	0.0059	0.0005	41.53	0.0099	0.0011	72.92

Genotype	C27	SE	Percent Reduction	C29	SE	Percent Reduction	C31	SE	Percent Reduction	Total Alkanes	SE	Percent Reduction
Sabine	0.0758	0.0083	0	0.1906	0.0095	0	0.2278	0.0062	0	1.6248	0.0312	0
6-1A	0.0237	0.0026	68.69	0.0502	0.0057	73.64	0.0231	0.0045	89.85	0.3110	0.0127	80.86
7-17A	0.0263	0.0028	65.27	0.0681	0.0074	64.24	0.0293	0.0041	87.14	0.3918	0.0149	75.89
11-39A	0.0286	0.0017	62.24	0.0332	0.0024	82.60	0.0572	0.0050	74.88	0.4142	0.0106	74.51

Supplementary Table S5 Summary of ketones measured, three replicates per genotype.

Genotype	C19	SE	Percent Reduction	C21	SE	Percent Reduction	C23	SE	Percent Reduction
Sabine	0.0002	0.0000	0	0.0002	0.0001	0	0.0004	0.0001	0
6-1A	0.0034	0.0003	-1,605.00	0.0078	0.0010	-3,459.09	0.0129	0.0014	-3,053.66
7-17A	0.0032	0.0001	-1,515.00	0.0073	0.0003	-3,209.09	0.0096	0.0007	-2,241.46
11-39A	0.0001	0.0000	30.00	0.0003	0.0001	-31.82	0.0003	0.0000	17.07

C3.in

Genotype	C25	SE	Percent Reduction	C27	SE	Percent Reduction	C29	SE	Percent Reduction	Total Ketones	SE	Percent Reduction
Sabine	0.0008	0.0001	0	0.0020	0.0003	0	0.0041	0.0004	0	0.0231	0.0008	0
6-1A	0.0135	0.0012	-1,697.33	0.0121	0.0011	-507.00	0.0061	0.0012	-48.18	0.1676	0.0061	-626.58
7-17A	0.0107	0.0006	-1,330.67	0.0101	0.0003	-405.00	0.0064	0.0002	-55.96	0.1421	0.0018	-515.78
11-39A	0.0007	0.0000	5.33	0.0010	0.0001	49.50	0.0027	0.0001	35.52	0.0154	0.0002	33.12

Supplementary Table S6 Summary of sterols observed; three replicates per genotype.

Genotype	C27	SE	Percent Reduction	C28	SE	Percent Reduction	C29	SE	Percent Reduction	Total Sterols	SE	Percent Reduction
Sabine	0.0010	0.0001	0	0.00001	0.0000	0	0.02196	0.0016	0	0.02296	0.0017	0
6-1A	0.0013	0.0002	-28.39	0.00209	0.0002	-20,760.84	0.01050	0.0013	52.18	0.01386	0.0015	39.63
7-17A	0.0011	0.0000	-10.44	0.00255	0.0002	-25,373.34	0.01179	0.0006	46.29	0.01543	0.0007	32.77
11-39A	0.0011	0.0003	-6.54	0.00001	0.0000	0.00	0.03378	0.0036	-53.86	0.03485	0.0033	-51.80

Measurement reported as $\mu g/cm^2$
SE: Standard Error
Percentage Reduction: Relative to wild-type ‘Sabine’
Fatty Acids: C16 – C32 Saturated Fatty Acids
Aldehydes: C22 – C34 Aldehydes
1° Alcohols: C20 – C34 Primary Alcohols
Alkanes: C21 – C31 Alkanes
Sterols: C27 0 C29 Sterols
Ketones: C19 – C29 Ketones
